# Epigenetically Mediated Pathogenic Effects of Phenanthrene on Regulatory T Cells

**DOI:** 10.1155/2013/967029

**Published:** 2013-03-07

**Authors:** Jing Liu, Luhua Zhang, Lisa C. Winterroth, Marco Garcia, Shannon Weiman, Jillian W. Wong, John B. Sunwoo, Kari C. Nadeau

**Affiliations:** Stanford University School of Medicine, Division of Immunology and Allergy, Grant Building, 3rd Floor, S370, MC5208, Stanford, CA 94305, USA

## Abstract

Phenanthrene (Phe), a polycyclic aromatic hydrocarbon (PAH), is a major constituent of urban air pollution. There have been conflicting results regarding the role of other AhR ligands 2,3,7,8- tetrachlorodibenzo-p-dioxin (TCDD) and 6-formylindolo [3,2-b]carbazole (FICZ) in modifying regulatory T cell populations (Treg) or T helper (Th)17 differentiation, and the effects of Phe have been understudied. We hypothesized that different chemical entities of PAH induce Treg to become either Th2 or Th17 effector T cells through epigenetic modification of FOXP3. To determine specific effects on T cell populations by phenanthrene, primary human Treg were treated with Phe, TCDD, or FICZ and assessed for function, gene expression, and phenotype. Methylation of CpG sites within the *FOXP3* locus reduced *FOXP3* expression, leading to impaired Treg function and conversion of Treg into a CD4^+^CD25^lo^ Th2 phenotype in Phe-treated cells. Conversely, TCDD treatment led to epigenetic modification of IL-17A and conversion of Treg to Th17 T cells. These findings present a mechanism by which exposure to AhR-ligands mediates human T cell responses and begins to elucidate the relationship between environmental exposures, immune modulation, and initiation of human disease.

## 1. Introduction

The aryl hydrocarbon receptor (AhR) is a ligand-activated member of the basic-helix-loop-helix family of transcription factors that acts as a sensor for a wide variety of environmental air pollutants, including polycyclic aryl hydrocarbons (PAHs) [1–3]. Recent studies suggest that AhR signaling also has a role in development and immune modulation (reviewed in [4]). Ligand-activation of AhR plays a role in the differentiation of mouse Treg and Th17 cells with implications in autoimmune disease pathogenesis; however, there is controversy as to whether specific AhR-ligands regulate murine Treg versus T helper (Th)17 cell differentiation [5–10]. TCDD has been shown to induce Treg in mouse models [5, 9]. Additional complicating factors for understanding the mechanism of AhR-ligand-mediated effects on T cell immune function include the tissue and species specificity, in addition to the ligand-specific differences in the structure, function, and mechanism of action of the AhR itself [6]. Currently, there is limited information as to how AhR ligands modulate human T cells, the expression of AhR by different human T cell subsets, and the mechanisms by which AhR agonists change human T cell populations. A fuller understanding of these mechanistic pathways and effects of AhR-ligands on human T cells could enhance our knowledge of the possible impact of environmental toxins on the immune response.

Previously, our group and others have shown that exposure to PAHs, a major constituent of urban air pollution [1–3, 11–20], is associated with decreased function of Treg via increased methylation of CpG sites in the forkhead box transcription factor 3 (FOXP3) locus. FOXP3 is important to Treg development and suppressive function [21], and its repression via CpG island methylation in promoter and intronic transcriptional regulatory regions results in transformation of Treg into effector T cells (Teff) [22–24]. Conversely, demethylation of these regions is associated with and appears to be required for stable FOXP3 expression in Treg [22, 25]. This demethylation is associated with conversion of Teff to Treg [22] and can be induced by inhibitors of DNA methyltransferases (DNMTs) [23].

We hypothesized that PAHs (specifically, phenanthrene) act via the AhR to mediate effects on several key downstream elements, including activation of DNMTs, ultimately leading to methylation of the *FOXP3 *gene, instability of FOXP3 expression, and impairment of Treg function. Given previous reports of FOXP3 instability mediating conversion of Treg to Teff in mouse models [26], we further postulated that our observed Treg impairment was due in part to the conversion of Treg to Teff cells (Th1, Th2, or Th17 cells). Recently, AhR has been shown to be important in the regulation of mouse T cell differentiation, and AhR was shown to be expressed in Th17 and Treg [5, 9]. Depending on the ligand used, AhR activation reciprocally regulated Treg versus Th17 cells. Whereas TCDD induced Treg, FICZ induced Th17 cells in mouse models [5, 9, 27]. Additionally, others have shown that activation of AhR by TCDD induces upregulation of Fas and Fas ligand, thereby promoting activation-induced cell death [23]. Therefore, we hypothesized that the mechanism of action of T cell modulation by phenanthrene was ligand specific and different from other AhR ligands like TCDD or FICZ in that Treg exposed to Phe could be switched to a Th2 phenotype. 

In summary, our findings confirm these hypotheses, indicating that *ex vivo* exposure of purified human Treg to Phe (a relatively stable ligand of AhR) [28] transforms the Treg into Th2 cells. This switch from Treg to Th2 cells was found to be due to increases in CpG island methylation of *FOXP3 *and subsequent decreases in FOXP3 expression in an AhR-, DNMT1-, and DNMT3b-dependent fashion. In contrast, treatment of human Treg with TCDD (a relatively stable ligand of AhR) [28] or FICZ (a relatively unstable ligand) [28] induced a Th17-like Teff phenotype. These mechanisms indicate how the regulatory T cell arm of the human immune system could be impaired by Phe exposure and have significant implications in the relationship between environmental exposures and human disease, such as atopic conditions.

## 2. Materials and Methods

### 2.1. Healthy Subjects

We defined healthy subjects (18–65 yrs) as nonsmokers with a total serum IgE of <25 IU/mL, negative skin testing as compared with positive histamine control, and no evidence of lung diseases or active infection. Subjects were excluded if there was evidence of chronic infection, or if they were currently taking medications (specifically any inhaled or oral steroid or any antiproliferative agent). This study was approved by the Stanford Administrative Panel on Human Subjects in Medical Research. All subjects signed consent forms as per good clinical practice (GCP) guidelines.

### 2.2. T Cell Culture

T cell isolation and phenotyping can be found in this paper's Supplemental Text (available online at http://dx.doi.org/10.1155/2013/967029).

Phenanthrene solution was purchased from Sigma-Aldrich (Supelco 40047, analytical standard, 5,000 ug/mL, ampule of 1 mL). 2,3,7,8-Tetrachlorodibenzo-p-dioxin (TCDD), 6-formylindolo[3,2-b]carbazole (FICZ) and 5-Aza-2′-deoxycytidine (decitabine) were purchased from Sigma. 3′,4′-Dimethoxyflavone (3′-4′-DMF) was purchased from Alfa Aesar. All cell culture solutions (including those with AhR ligands) were changed daily. Cells were treated with either 300 nM TCDD, 300 nM FICZ, or 300 nM phenanthrene for 24, 48, 72, or 168 hours in the presence of 30 IU/mL IL-2. Diluent for the polycyclic aromatic hydrocarbon compounds was at a final concentration of no more than 0.1% DMSO. Also, 10 uM 3′,4′-DMF or 1 uM decitabine was added in combination with phenanthrene at *T* = 0 for AhR/DNMT inhibitor experiments. In reversibility assays, phenanthrene was washed out 1, 3, or 4 days after incubation and cultured for the remainder of 7 days in phenanthrene-free culture media. Cytokine rescue assays were performed by adding 10 ng/mL of cytokine (Miltenyi, BD Biosciences) in conjunction with phenanthrene at *T* = 0 and replaced after phenanthrene wash out. In IL-2 experiments, additional cytokine was added to base level IL-2 present in RPMI. Samples were run in duplicate.

### 2.3. Methylation and Transcriptional Analysis

Genomic DNA was prepared from purified Treg or Teff using a standard blood DNA extraction kit (Qiagen). DNA was denatured, modified with sodium metabisulfite, purified, and desulfonated using a CpGenome Fast DNA modification kit (Chemicon International).

Disulfide oligonucleotide primers were designed using MethPrimer software.

For FOXP3, 13 CpG sites were sequenced (6 in promoter and 7 in intron):

 FOXP3-promoter-CpGs-Forward: TATAATTAAGAAAAGGAGAAATAT-AGAGAG
 FOXP3-promoter-CpGs-Reverse: TCAACCTAACTTATAAAAAACTATCAC
 FOXP3-intron-CpGs-Forward: TTGGGTTAAGTTTGTTGTAGGATAG
 FOXP3-intron-CpGs-Reverse: ATCTAAACCCTATTATCACAA.

For IL-17A, 6 CpG sites were sequenced (6 in promoter):

 IL-17A-promoter-CpGs-Forward TTTGATACGGGAAGTTTTTTC
 IL-17A-promoter-CpGs-Reverse ATTTCGTAGGGGATTTACGTA.

DNA was then sequenced and analyzed by SayoBiotech (Palo Alto, CA). 

Quantitative real-time (QT-PCR) was performed for FOXP3, AhR, DNMT1, and DNMT3b and analyzed by published methods [29]. All primers were designed using NCBI Primer-Blast search and/or published. [29]. All transcripts were normalized to *β*-glucuronidase. Samples were run in duplicate. See Table 1.

### 2.4. Treg Suppression, Proliferation, and Chemotaxis Assays

Treg and Teff cells were used in standard 3H thymidine proliferation assays and chemotaxis assays according to published methods (Promocell). [6, 12, 26, 29]. A stimulation index value (% suppression) was calculated from duplicate Treg suppression assays [6, 12, 15, 16]. Chemotaxis indices were generated from number of Treg migrating to primary HBEC (Promocell) divided by number of spontaneously migrated cells, as previous published [12]. Samples were run in duplicate.

### 2.5. Mouse Studies

AhR+/− mice from Jackson Laboratory were bred to produce AhR+/+, AhR+/−, and AhR−/− mice and were used for experiments between 6 and 12 weeks of age. Details can be found in this paper's Supplemental Text.

### 2.6. Statistical Analysis

Our laboratory performed statistical analysis using the GraphPad Prism Software (Version 5.0). Spearman correlation coefficients for nonparametrically distributed data are shown. Differences were assessed using one-way ANOVA non parametric Kruskall-Wallis test and pairwise posttest comparisons via Dunn's multiple comparison test. *P* values ≤0.05 were considered significant. Error bars represent standard deviation from the mean.

## 3. Results

### 3.1. Treg Exposed to Phenanthrene Demonstrate Increased Methylation in the *FOXP3* Locus and Decreased *FOXP3* Expression

**Figure 1:**
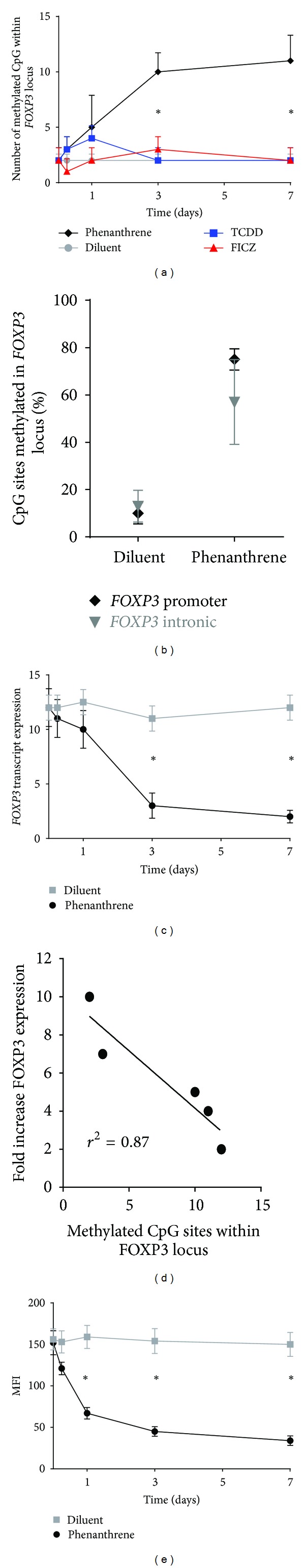
Phenanthrene exposure increases CpG methylation and reduces FOXP3 expression in primary Treg. Purified CD4^+^CD25^hi^CD127^lo^ FOXP3+ Treg from PBMC of healthy donors (*n* = 5) were incubated with 300 nM Phe (black), TCDD (blue), FICZ (red), or diluent (grey) for 0 to 7 days. (a) Quantification of methylated CpG sites (out of 13 total promotor and intronic sites) within the FOXP3 locus over the 7-day culture. (b) After a 2-day incubation with Phe, DNA was analyzed for percentage of methylated CpG sites within promoter (black square, *n* = 6) and intronic (gray triangle, *n* = 7) sequences of the *FOXP3* locus. (c) *FOXP3 *transcripts were quantified with QT-PCR and normalized to the housekeeping gene **β*-glucuronidase*. (d) Linear regression analysis of *FOXP3* transcript expression to the number of methylated CpG sites within the *FOXP3* locus; *r* = Spearman's correlation coefficient. (e) Intracellular FOXP3 protein expression was measured by flow cytometry and reported as median fluorescence intensity (MFI). *P < 0.05; error bars represent SD.

We hypothesized that Phe, a semivolatile PAH and major component of urban air pollution [1–3, 11–20], modulates the function of Treg through methylation of CpG regions within the *FOXP3 *locus. To test this hypothesis, peripheral blood mononuclear cells (PBMCs) were isolated from healthy human donors (*n* = 5), and CD4^+^CD25^hi^CD127^lo^ Treg were further purified by flow sorting [28–31]. After 0 to 7days of incubation with 300 nM Phe, we examined methylation of 13 distinct CpG sites in promoter (*n* = 6) and intronic (*n* = 7) regions of the *FOXP3 *gene according to previously published methods [29]. We found that Phe exposure resulted in significant increases in CpG methylation within the FOXP3 locus over the 7-day culture (Figure 1(a)). In contrast, treatment of Treg with TCDD or FICZ did not modify CpG methylation of FOXP3 over the 7-day culture, and treatment of Treg with TCDD led to an increase in CpG methylation within the IL-17A promoter at day 7 of culture (Supplemental Figure  S1). Increases in FOXP3 CpG methylation were observed in both promoter and intronic regions of *FOXP3* over time. After 7 days, up to 80% of promoter and 75% of intronic enhancer CpG sites assessed were methylated in Phe-treated Treg versus 20% and 15%, respectively, in media only treated controls (Figure 1(b)). Consistent with the increase in CpG methylation of *FOXP3*, FOXP3 expression was reduced over time in Treg treated with Phe. Increases in *FOXP3 *methylation corresponded to a 2.8-fold reduction in *FOXP3 *transcription by day 7 compared to Treg treated with diluent (Figure 1(c)) and was directly associated with lower FOXP3 expression (Figure 1(d)). Finally, treatment of Phe resulted in a 2.5-fold reduction in FOXP3 protein expression as early as 1 day of treatment in culture (Figure 1(e)). In summary, Phe, but not TCDD or FICZ, induced methylation changes in the *FOXP3 *locus, leading to decreased transcript and protein expression of FOXP3 throughout the time course of exposure *ex vivo*.

### 3.2. Impaired Function in Treg after *Ex Vivo* Phenanthrene Exposure

**Figure 2:**
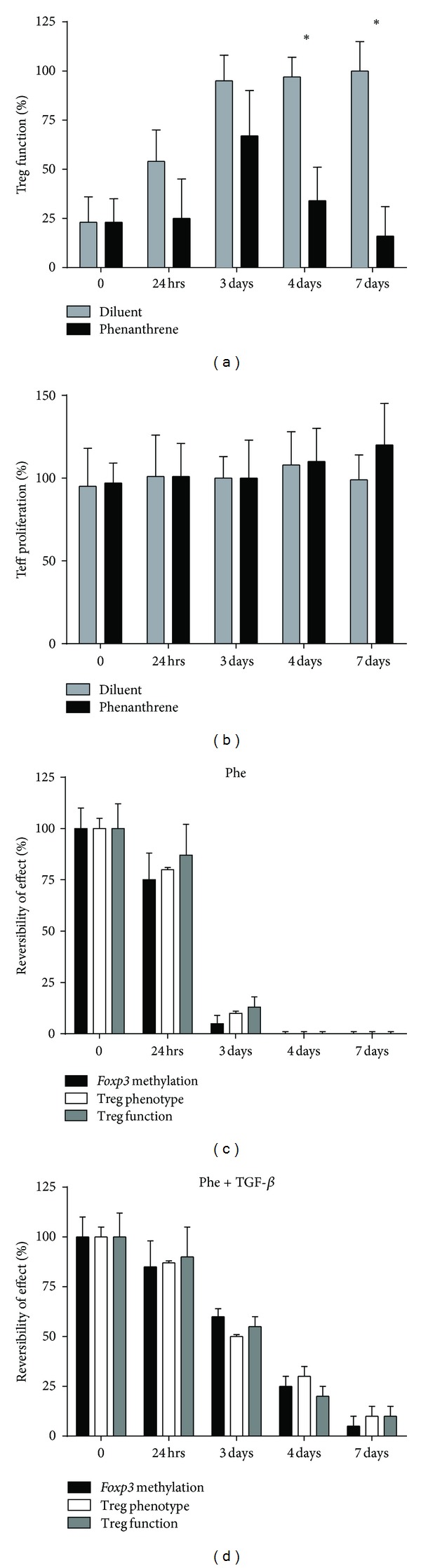
Phenanthrene exposure irreversibly impairs Treg function. (a) Isolated Treg from individual healthy donors (*n* = 5) were incubated with 300 nM Phe (black bars) or diluent (grey bars) for 0 to 7 days and analyzed for function using standard Treg suppression assays. (b) Teff proliferation was analyzed in Phe or diluent treated cultures of isolated Teff without Treg. To test the irreversibility of effects of Phe on Treg, Treg cultures were treated with Phe (c) or Phe+TGF*β* (d) on day 0, followed by a wash and replacement with Phe-free media (+/−TGF*β*) on either day 1 (24 h), 3, 4, or 7. CpG methylation of the *FOXP3* locus (*n* = 13 CpG sites, 6 in promoter region, 7 in intronic region) (black bars), Treg phenotype (white bars), and Treg function (grey bars) of cultured Treg were assessed on day 7. *P < 0.05; data represents mean +/− SD.

Given that FOXP3 has been shown to be important to Treg function, our findings that Phe decreased FOXP3 expression led us to hypothesize that exposure of Treg to Phe would also lead to Treg dysfunction. Using autologous T cell cultures from healthy donors controlled for cell numbers, we performed standard functional assays for Treg suppression and proliferation [28–31] using Treg exposed to Phe for different time points. We observed significant decreases in Treg function on days 4 to 7 of Phe treatment relative to Treg treated with diluent control (Figure 2(a)). While initial Treg populations were purified to over 97% purity, we cannot rule out the possibility that the few remaining Teff cells could have confounded functional and phenotypic assays. To control possible increased Teff proliferation in response to Phe treatment, we performed Teff proliferation assays in parallel conditions and did not find significant differences in proliferation that could account for the profound impairment in Treg function (Figure 2(b)). Thus, *ex vivo* treatment of Treg with Phe leads to decreased FOXP3 expression and a subsequent decrease in Treg suppressor function.

### 3.3. Early Effects of Phenanthrene Exposure on Treg Are Irreversible

We examined the reversible nature of Phe's effects by washing out Phe on day 0, 1, 3, 4, or 7, followed by incubation in Phe-free media for the remainder of the time course. Functional, methylation, and immunophenotyping studies were performed on day 7. Results indicate that reversibility of all effects was dramatically abrogated after 3 days of coculture with Phe (Figure 2(c)). We next tested whether reversibility could be rescued by administration of TGF-*β*, IL-10, or IL-2, which have been found to stabilize Treg phenotype in *ex vivo* cultures [23, 32, 33]. Cytokines were added in conjunction with Phe at the initiation of cultures, followed by wash-out of Phe, and replaced for the remainder of the time course. Functional, methylation, and immunophenotyping studies were performed on day 7. We found that TGF-*β* was able to partially reverse methylation of the *FOXP3 *gene, with improved function and maintenance of Treg phenotype (Figure 2(d)). No effects on reversibility were documented after incubation with IL-10, IL-2, IL-6, or IL-21 (Supplemental Figures S2(a)–S2(d)).

### 3.4. Phenanthrene Exposure Results in an Unstable Treg Population That Converts to a Teff Phenotype

**Figure 3:**
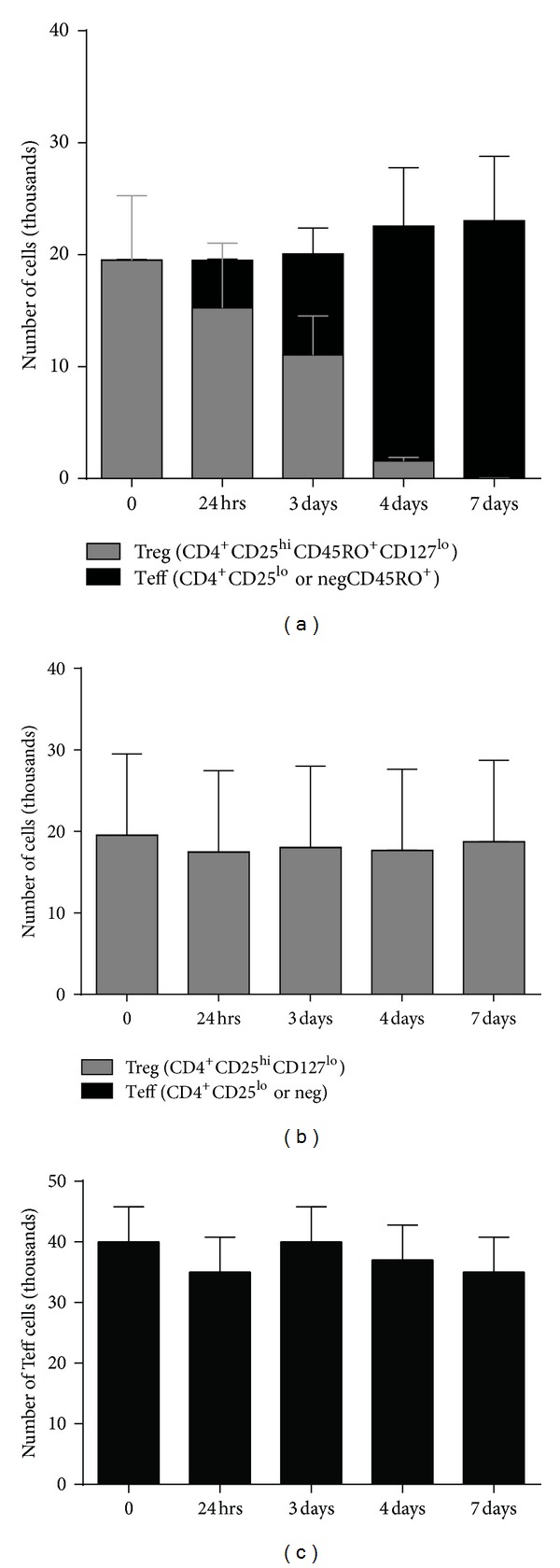
Phenanthrene exposure results in Treg to Teff conversion. Isolated Treg from individual healthy donors (*n* = 5) were incubated with 300 nM Phe for 0 to 7 days and analyzed for T cell phenotype. Cells were analyzed at 5 time points by immunophenotyping for Treg (CD4^+^CD25^hi^CD127^lo^, gray bars) and Teff (CD4^+^CD25^neg/lo^, black bars) subsets in cultures exposed to Phe (a) versus diluent alone (b). Teff incubated with Phe did not show decreases in cell number over time (c). Error bars show SD.

We next hypothesized that Phe, through modulations of FOXP3 (Figure 1), could lead to Treg conversion to Teff. We used immunophenotyping of cultured Treg from healthy donors to analyze conversion of Treg (CD4^+^CD25^hi^CD127^lo^  CD45RO^+^) to Teff (CD4^+^CD25^neg/lo^ CD45RO^+^) during exposure to Phe. We observed a conversion of Treg to Teff in as little as 24 hours of exposure to Phe (Figure 3(a)) compared to Treg treated with diluent alone (Figure 3(b)). Again, to control Teff proliferation as a confounding factor, we incubated Teff cells alone (without Treg present) with Phe and found Teff proliferation to be insufficient to account for Teff numbers observed in the conversion assay (Figure 3(c)).

### 3.5. Phe Converts Treg to Th2, Whereas TCDD and FICZ Convert Treg to Th17 Cells

**Figure 4:**
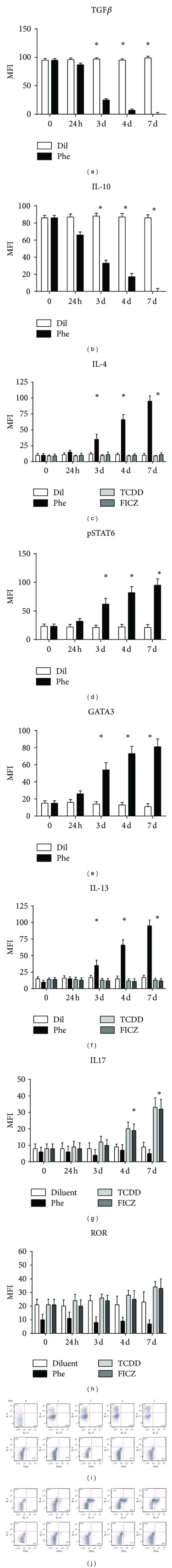
Phe induces conversion of Treg to Th2 Teff. Isolated Tregs were treated with 300 nM Phe (black bars), TCDD (light grey), FICZ (dark grey), or diluent (white bars) for 0 to 7 days and analyzed for cytokine production by immunostaining and flow cytometry. MFIs (×10^3^) are represented on the *y* axes. Th2 Teff phenotype was identified by decreased (a) TGF-*β* and (b) IL-10 production and increased IL-4 (c), pSTAT6 (d), GATA-3 (e), and IL-13 (f), and Th17 phenotype was examined by IL-17 (g) and ROR-*γ*-T (h) production. Representative flow cytometry plots of IL-4+ or IL-17+ T cell populations at each time point for Treg treated with Phe (i) or TCDD (j). *P < 0.05; error bars show SEM.

To better define the T cells induced in the Phe-treated cultures, the CD4^+^CD25^neg/lo^ subset of Teff that was induced by Phe from the CD4^+^CD25^hi^CD127^lo^ Treg population was further analyzed for cytokine production by immunostaining and flow cytometry (Supplemental Figure  S3). Phe-exposed T cells showed decreased TGF-*β* and IL-10 and increased IL-4, IL-13, tyrosine-phosphorylated (p)STAT6, and GATA-3 over time relative to cells treated with diluent control (Figures 4(a)–4(f) and 4(i)) and consistent with a Th2 Teff phenotype [34]. No alterations were documented in T-bet, IL-12, or IFN-*γ* production, indicating lack of Th1 induction [20] (Supplemental Figures S4(a)–S4(c)). IL-17 and ROR-*γ*t production also did not change over time with Phe exposure, ruling out possible Th17 induction [3, 34]. However, in contrast, treatment of Treg with TCDD or FICZ induced a T cell population which expressed IL-17 and ROR-*γ*-T after 7 days of culture (Figures 4(g)–4(j)). IL-9, IL-22, or IL-21 production was also assessed to evaluate whether additional Teff subsets [3, 34] were induced, but no significant changes were documented in these cytokines over the time course of the experiments (Supplemental Figures  S4(d)–S4(f)). To summarize, treatment of Treg with TCDD or FICZ induced a Th17 phenotype, whereas Phe induced a Th2 phenotype after 7 days of culture.

### 3.6. AhR Found to Be Increased in Expression in Th17 and Treg Cells

**Figure 5:**
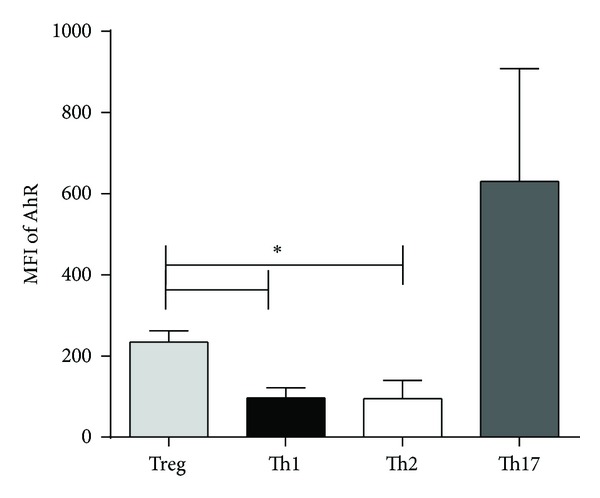
Expression of AhR on T cell subsets. Isolated PBMCs (*n* = 5 individual subjects) were stained with fluorescently conjugated AhR antibodies, gated for Treg, Th1, Th2, or Th17 cell subsets and simultaneously assessed for AhR expression (as shown by median fluorescence intensity (MFI)). Error bars show SD; *P < 0.05.

We next assessed the baseline level of AhR protein expression in T cell subsets taken from freshly isolated PBMC to understand whether human Treg displayed increased susceptibility to Phe compared to that of Th2, Th1, or Th17 human cells. Intracellular labeling with directly conjugated fluorescent antibodies to AhR, to capture both intracellular and surface AhR, demonstrated that AhR expression in Treg was nearly 2.5 times that of AhR expression in Th2 indicating that AhR ligands, such as Phe, may have a greater impact on Treg and Th17 populations as compared to Th1 or Th2 populations (Figure 5).

### 3.7. T Cell Immune Deviation due to Phe Is not Associated with Apoptosis or Cell Death Pathways

Since the toxicity of PAHs may be attributed in part to induced cell death and apoptosis [35], we next determined absolute numbers of live and dead cells and markers of apoptosis throughout the time course of the *ex vivo* experiments. We did not observe increases in cell death via propidium iodide staining, annexin V staining, or caspase 8 transcript expression in either Treg or Teff populations with treatment of Phe (Supplemental Figures  S5(a) and S5(b)).

### 3.8. T Cell Migration to Epithelial Cells Is Impaired due to Pheexposure

In addition to examining cytokine production associated with T cell subtypes (i.e., Th1, Th2, Th17, Th9, and Treg), we also determined whether chemokine molecules shown to be important for Treg migration in human and murine models, CCR4 and CCR8, [29, 36–40] were modulated by Phe exposure. Since FOXP3 has been shown to control transcription of chemokine receptors CCR4 and CCR8 [36–40], we hypothesized that CCR4 and CCR8 would be decreased over time after exposure to Phe. Significant decreases in CCR8 and to a lesser extent CCR4 protein expression were observed, with CCR8 dropping 97% and CCR4 dropping 30% from day 0 to day 7 of culture in Treg treated with Phe relative to Treg treated with diluent (Supplemental Figures  S6(a) and S6(b)). As both chemokine receptors have been implicated in Treg homing to relevant tissues and function in moderating inflammation [30, 38], we utilized chemotaxis assays to assess the migratory potential of Treg treated with Phe versus Treg incubated with diluent. A 5-fold decrease in chemotaxis of Treg to primary bronchoepithelial cells was observed after 24 hrs of incubation with Phe (Supplemental Figure  S6(c)). At this time, CCR8 and CCR4 expression dropped by 35% and 12%, respectively, indicating that longer periods of exposure of Treg to Phe would likely show even greater deficits in chemotaxis. Collectively, these data suggest that treatment of Treg with PAH contributes to Treg dysfunction by converting Treg populations into Teff and essentially depleting Treg suppressive populations, as well as modifying chemokine receptors important for chemotaxis.

### 3.9. Modulation of Human Treg by Phenanthrene Occurs through the AhR and Involves DNMT1 and DNMT3b

**Figure 6:**
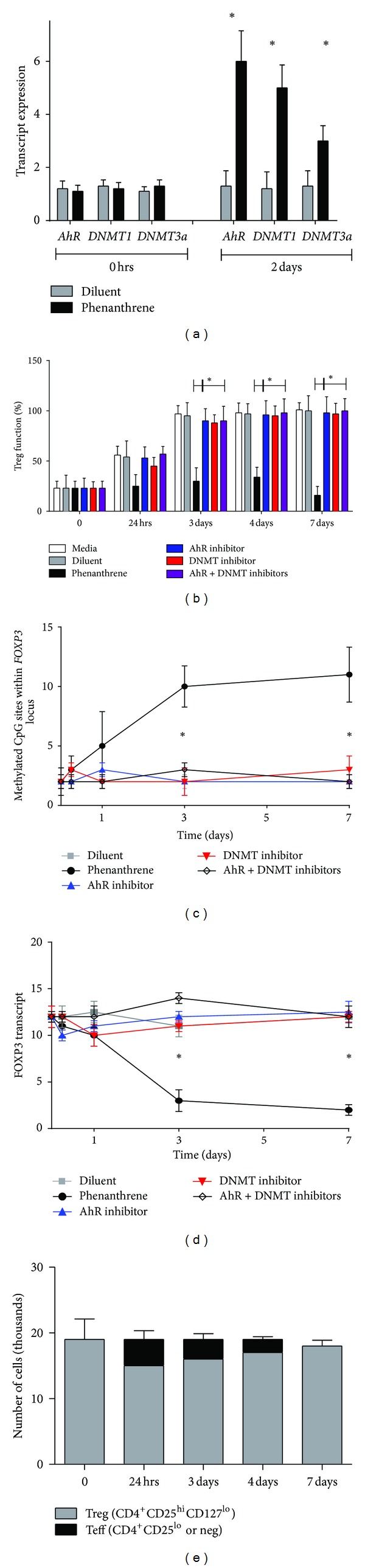
AhR, DNMT1, and DNMT3b mediate Phe's effects on Treg. (a) Treg purified from healthy donors (*n* = 5) were treated with 300 nM Phe (black bars) or diluent (grey bars) for 2 days followed by quantification of *AhR*, *DNMT1,* and *DNMT3b* transcript by QT-PCR. Treg function, (b) *FOXP3* locus methylation (c), and *FOXP3* transcript (d) were assessed in 7-day Treg cultures treated with diluent (grey) and Phe (black) or co-incubation with Phe and the DNMT inhibitor decitabine (1 uM; red), AhR inhibitor 3′,4′-dimethoxy-flavone (10 uM; blue) or both inhibitors (purple). (e) Conversion of Treg to Teff in 7-day cultures treated with Phe + 3′DMF.

To determine the molecular mechanism by which Phe leads to DNA methylation in Treg, we tested the possibility that Phe might directly enhance expression of DNMT1 and 3b, which are candidate methyltransferases involved in maintenance and *de novo* CpG island methylation, respectively [41–43]. Expression analysis using QT-PCR 2 days after Phe treatment on purified Treg from healthy control samples (*n* = 5) showed increased levels of *DNMT1*, *DNMT3b,* and *AhR* transcripts (Figure 6(a)), suggesting a potential role for DNMT in alteration of *FOXP3* methylation. We next tested whether blocking DNMT activity with the known inhibitor, decitabine, would prevent Phe-induced effects. Coincubation of Treg with decitabine abrogated Phe's effects on Treg functional impairment (Figure 6(b)), *FOXP3* methylation (Figure 6(c)), FOXP3 downregulation (Figure 6(d)), and phenotypic transformation to Teff cells (Figure 6(e)) as early as day 3 of culture. These data demonstrate that Phe-induced Treg impairment is dependent upon DNMT in human T cells.

We subsequently examined upstream factors in the signal transduction cascade, focusing on the AhR, which has been reported to bind PAHs, translocate to the nucleus, and activate transcription of specific target genes [44]. AhR inhibition via pharmacologic intervention using a well-defined antagonist 3′,4′-dimethoxy-flavone (3′,4′-DMF) [45] blocked phenanthrene's effects on Treg functional impairment (Figure 6(b)), *FOXP3* locus methylation (Figure 6(c)), *FOXP3* transcription (Figure 6(d)), and phenotypic transformation to Teff cells (Figure 6(e)). Inhibition of both AhR and DNMT resulted in similar reversibility, and cotreatment did not result in compounding effects, implying that AhR activation and DNMT are both involved in phenanthrene-mediated Treg dysfunction.

Finally, we briefly investigated the *in vivo* role of AhR and DNMT as intermediate molecular links in the effect of Phe on FOXP3 regulation and Treg conversion with mouse immune cells. Teff and Treg from splenocytes of untreated AhR +/+, AhR +/−, and AhR −/− mice were assessed for *Dnmt1*, *Dnmt3b*, *Foxp3,* and *AhR* transcripts. As predicted from our human Treg data in which activation of AhR led to decreased FOXP3 expression, lack of AhR in mice was associated with significant increases in *Foxp3* and reduced *Dnmt1* and *Dnmt*3b expression in both Teff and Treg as compared to AhR +/− or AhR +/+ mice (Figures  S7(a) and S7(b)). 

In summary, our data indicate that the impact of Phe on *FOXP3* methylation and expression in human Treg is highly dependent on AhR activation and AhR-mediated effects on DNMT1 and 3b expression.

## 4. Discussion

These studies demonstrate that in *ex vivo* human studies, Phe activates the AhR pathway, altering CpG methylation patterns in Treg. Increased CpG methylation in key regulatory regions of the *FOXP3* gene destabilizes FOXP3 expression, resulting in the conversion of Treg to the proallergic Th2 Teff phenotype. Our studies indicate that *ex vivo* AhR activation via Phe and downstream events resulting in Treg to Th2 conversion are AhR ligand specific. These data demonstrate the important role of Phe in T cell plasticity and pathophysiology of atopic diseases [3, 32, 33, 46].

Recently, others have shown that Treg convert to Th2 [47] in culture; however, in contrast to these previous findings, our data demonstrate that specific environmental insults are needed to induce Treg deviation to Th2 or Th17 phenotypes. In our hands, we did not observe spontaneous conversion of Treg to Th2. The work presented herein is the first to our knowledge to present a detailed mechanism for how Treg conversion to other T cell phenotypes occurs via Phe activation *ex vivo*. Our findings provide insight into one mechanistic explanation of PAH-induced modulation of Treg function. We understand that there are several limitations with *ex vivo* data, including, but not limited to, the dose of Phe, a type of PAH, and PAH metabolite actually present *in vivo,* as well as the involvement of other immune cells and gene loci in the PAH response.

AhR has been shown to modulate Treg differentiation in a ligand specific fashion [5, 6]. TCDD, an exogenous nondegradable high-affinity AhR ligand, induces functional Treg cells that suppressed experimental autoimmune encephalomyelitis [5]. In contrast, FICZ, an endogenous rapidly degradable high-affinity AhR ligand, interferes with Treg cell development, boosts Th17 cell differentiation, and increases the severity of disease [5]. We also tested the TCDD and FICZ ligands and found that both increased human Th17 differentiation *ex vivo* without altering FOXP3 methylation (Figures 1 and 4). These results further support ligand-specific effects. FICZ has been shown in some cases to promote Th17 differentiation and in some cases not [3, 48–51]. These discrepancies between studies may reflect differences in the mouse versus human systems [52] and/or the involvement of other factors that play a role in skewing Th17/Treg/Th2 development, such as retinoic acid or TGF-*β* production by dendritic cells [3, 48]. Our studies with Phe further support the ligand specific role of AhR in modulating Treg development and its dynamic plasticity while presenting a novel pathway of Treg instability in the human system.

Mice and humans exhibit significant differences in innate and adaptive immunity, including balance of leukocyte subsets, T cell signaling pathway components, and Th1/Th2 differentiation [52]. This may account for discrepancies in the recent literature debating whether AhR activation leads to Th17, Treg, or other T cell subset differentiation [5, 49–51, 53–56], as well as between our own *ex vivo* human assays and *in vivo* mouse studies. While our comparison of AhR+/+ to AhR-deficient mice suggests that AhR plays a similar role in regulating DNMT and FOXP3 expression in humans and mice Figures S7(a) and S7(b), differences in mouse versus human ligand binding to AhR continue to be an area of important investigation.

We recognize that while our data point to a possible mechanism involved in Phe-induced Treg conversion to a pro-allergic Teff phenotype (Th2-skewed) in humans, other AhR-ligands could mediate their effects through different mechanisms. A previous study demonstrated that TCDD-induced AhR activation ameliorates inflammation in the mouse colitis model [27]. Our results indicate that AhR activation leading to Treg to Th2 differentiation may not always be beneficial; during atopic conditions, ligand-specific AhR activation may be detrimental. Duration of AhR activation may also play a role. Persistent ligands that are resistant to degradation, such as TCDD, may induce negative feedback mechanisms that alter the AhR-mediated response [3, 56]. Our studies provide the basis for further work investigating (i) stability effects of natural and exogenous AhR ligands and (ii) differences in mice that elucidate Phe- and AhR-mediated pathophysiologies.

Although the current study does not address Phe-specific effects and downstream consequences in diseased (atopic) individuals, our previous studies and those from others have reported associations between PAH exposure, increased methylation of certain genes, impaired Treg function, and disease in humans [29, 57]. The current data strengthen these observations by demonstrating a causative relationship in *ex vivo *studies of Treg from healthy individuals and further elucidated the process by identifying genes involved and delineating a mechanistic pathway by which a PAH alters Treg function irreversibly within 3 days *ex vivo*.

A number of studies have shown that deficit in Treg cell function is associated with increasing severity of asthma [29–31, 58]. Our findings suggest that this may be due in part to alterations in CCR8 expression, which plays a key role in T cell homing to lung parenchyma and bronchial epithelium and has been implicated as an important factor in allergic inflammation and normal immune homeostasis in asthma [36–40]. By decreasing CCR8 expression in Treg, PAHs may limit chemotaxis to lung tissue, where regulatory T cells temper asthmatic and allergic responses. In addition, predominance of Th2-derived IL-4 and IL-13 could induce isotype switching to IgE, which is associated with allergic conditions [59]. Lack of Treg migration, isotype switching, and Treg to Th2 conversion may all contribute to increases in inflammation and atopic disease.

Our study has important implications for the future of immunomodulatory therapies. Epigenetic changes, particularly aberrant methylation of CpG islands in regulatory sequences of FOXP3, may be prevented or reversed by dynamic DNA remodeling enzymes. Alternatively, pathway targets such as AhR and DNMT may be inhibited by small molecules or other pharmacologic means if specific to Treg cells. Future studies identifying other key players in the conversion of Treg to Teff cells are needed to further understand the mechanism of AhR-ligand-mediated immune changes. Such understanding could lead to improvements in prevention of and treatment for immune-mediated diseases.

## Supplementary Material

T Cell Isolation and Phenotyping Healthy donor samples were obtained from the Stanford Blood Center and sorting via flow cytometry was performed (FACS Aria, BD Biosciences). Each population obtained from flow cytometry was tested for purity based on multicolor flow cytometry staining for CD4^+^CD25hiCD127lo (Treg) and CD4^+^CD25neg (conventional CD4^+^ T cell). Only samples meeting or exceeding 97% pure were used for further experimentation and analysis. Fluorescently labeled, directly conjugated antibodies were used. Further descriptions are provided in the supplementary material.

## Figures and Tables

**Table 1:** 

	Forward 5′-3′	Reverse 5′-3′
Foxp3	5′-gcctcctcttcttccttgaa-3′	5′-gtgaggctgatcatggct-3′
AhR	5′-cgctgaaacatgagcaaattgg-3′	5′-acagcttaggtgctgagtcacagg-3′
DNMT1	5′-gaggaagctgctaaggactagttc-3′	5′-actccacaatttgatcactaaatc-3′
DNMT3b	5′-tacacagacgtgtccaacatgggc-3′	5′-ggatgccttcaggaatcacacctc-3′
