# Supplementary material for: Epigenetically Mediated Pathogenic Effects of Phenanthrene on Regulatory T Cells
Source: J Toxicol. 2013 Mar 7;2013:967029. doi: 10.1155/2013/967029 (PMC3606805; doi:10.1155/2013/967029)

**Supporting Information**

**Materials and Methods**

*T Cell Isolation and Phenotyping*

Healthy donor samples were obtained from the Stanford Blood Center and peripheral blood mononucleocytes (PBMC) were isolated from over a ficoll gradient. PBMC were plated out in 12-well plates in RPMI supplemented with 10% fetal bovine serum, penicillin/streptomycin (1:100 dilution from stock solution), and human recombinant IL-2 (30IU/ml).

All PBMC samples were first subject to live/dead staining (Molecular Probes/Invitrogen). To isolate Treg, we stained the cells for CD4 (clone SK-3, BD Biosciences), CD25 (clone 4E3, Miltenyi Biotec), and CD127 (clone SB 199; BioLegend). Cells were sorted by flow cytometry for live $CD4^{+}CD25^{hi}CD127^{lo/-}$ Treg cells (FACS Aria, BD Biosciences). CD127 positive cells were excluded to ensure separation of activated convention T cells from stable Treg populations, allowing for the isolation of highly enriched $FOXP3^{+}$ cells. Live non-Treg, referred to as conventional effector T cells in this study, were simultaneously flow sorted by identification of $CD4^{+}CD25^{neg}$ populations. Each population obtained from flow cytometry was tested for purity based on multicolor flow cytometry staining for $CD4^{+}CD25^{hi}CD127^{lo}$ (Treg) and $CD4^{+}CD25^{neg}$ (conventional $CD4^{+}$ T cell). Only samples meeting or exceeding 97% pure were used for further experimentation and analysis.

Fluorescently labeled, directly conjugated antibodies (BD Biosciences, BioLegend or Thermo Scientific) against IL-17, IL-10, IL-4, IL-13, Foxp3, CD4, CD25, IL-21, CD194, T-bet, GATA-3, IFNγ, pSTAT6, IL-12, and RORγT were used to detect intracellular and

extracellular markers to differentiate Th17, Th1, Th2, and Treg. Intracellular cytokine and protein staining was performed as per the standardized protocol from BD Biosciences and as previously published. Data were acquired with a FACSCalibur or LSRII digital flow cytometer (BD Biosciences) through the Stanford Shared FACS facility; it was equipped with 4 lasers (405, 488, 535, 633 nm), 2 light scatter detectors (yielding forward and side scatter data) and 18 fluorescent detectors. We controlled acquisition using the DiVa software (BD Biosciences). FlowJo software was used for analysis (Treestar). Samples were run in duplicate.

*Analysis of Cell Death and Apoptosis.*

We performed live/dead staining (Molecular Probes/Invitrogen) and flow cytometric analysis of cells from each sample. PI/Annexin (BD Biosciences) staining and Caspase 8 transcript expression (Applied Biosystems) were performed according to manufacturer guidelines and standard protocol. Samples were run in duplicate.

*AhR-/- Mice*

Spleens were harvested and made into single cell suspensions. Cells were stained with antibodies against CD3, CD4, and CD25 (BD Pharmingen). CD4+ Teffs and CD3+CD4+CD25hi Treg were analyzed and sorted on a BD FACSAria. RNA was isolated using an RNeasy Mini Kit (Qiagen) and reversed transcribed with a SuperScript III First-Strand Synthesis Kit (Invitrogen). The transcripts were then amplified using a 7900HT Fast Real-Time PCR System and Taqman Gene Expression Assays (Applied Biosystems) against Dnmt1, Dnmt3b, Foxp3, AhR and housekeeping gene Hprt.

**Figure S1. Increases in CpG methylation within the IL-17A promotor in Treg after treatment with TCDD.** Treg cultures (n=5) were treated with 300nm TCDD (black bars) or diluent (grey bars) and 6 CpG methylation sites within the IL-17A promotor were assessed on day 7 of culture.

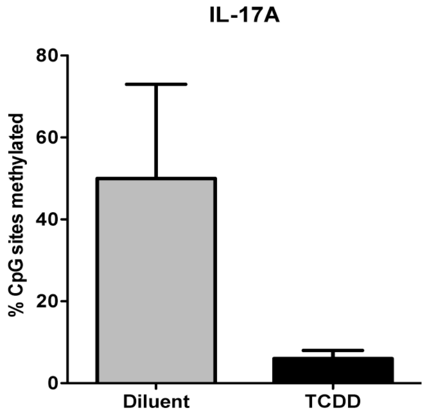

**Figure S2. Cytokine treatment does not reverse effects of Phe on Treg cultures.** Treg cultures were treated with Phe + IL-10 (A), + IL-2 (B), + IL-6 (C) or + IL-21 (D) on day 0, followed by a wash and replacement with Phe-free media + indicated cytokine on either days 1 (24 h), 3, 4, or 7. CpG methyation of the *FOXP3* locus (black bars), Treg phentoype (white bars) and Treg function (grey bars) of cultured Treg was assessed on day 7. Data represents mean +/- SD.

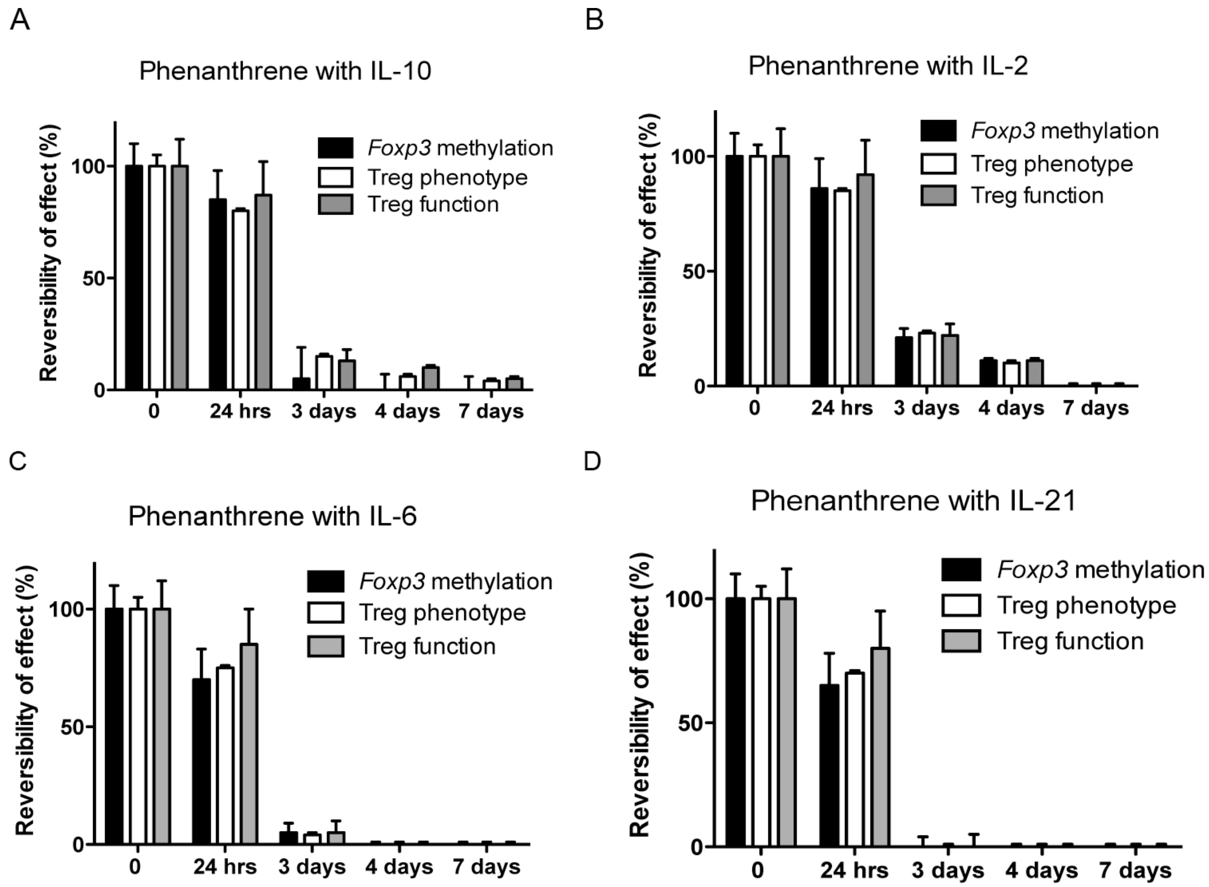

**Figure S3. Gating strategy for identifying Treg.**

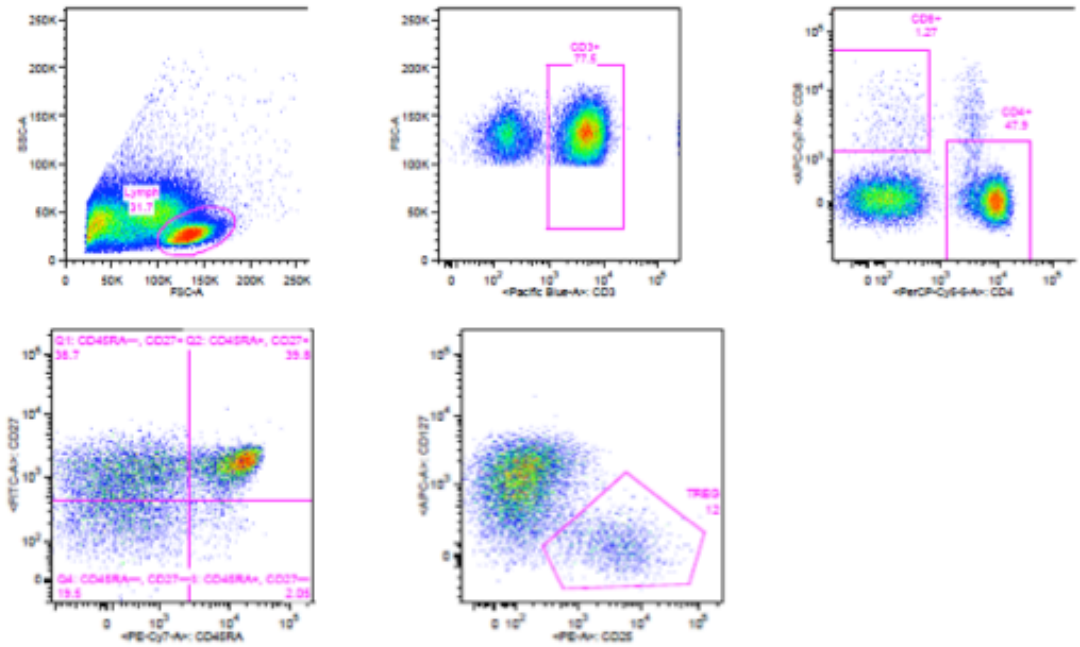

**Figure S4. Intracellular and extracellular flow cytometry studies reveal Th2 Teff conversion, and not Th1 conversion, by Phe.** Isolated Tregs were treated with 300 nM Phe (black bars), TCDD (light grey), FICZ (dark grey) or diluent (white bars), for 0 to 7 days and analyzed for cytokine production by immunostaining and flow cytometry.

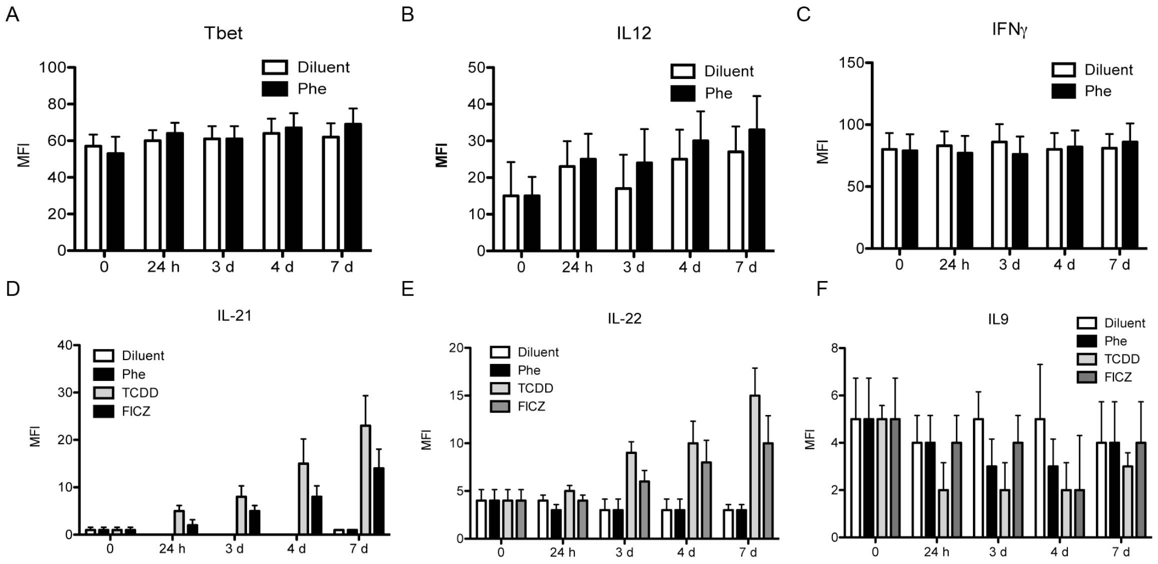

**Figure S5. Cell death and apoptotic markers are unaltered in both Treg and Teff treated with phenanthrene.** Tregs were cultured in the presence or absence of Phe as previously described. On day 7, cultures were analyzed by propidium iodide (circles), annexin 5 staining (gray downward triangles) and caspase (black upward triangles) and shown as percent of cells positive and error bars show SD.

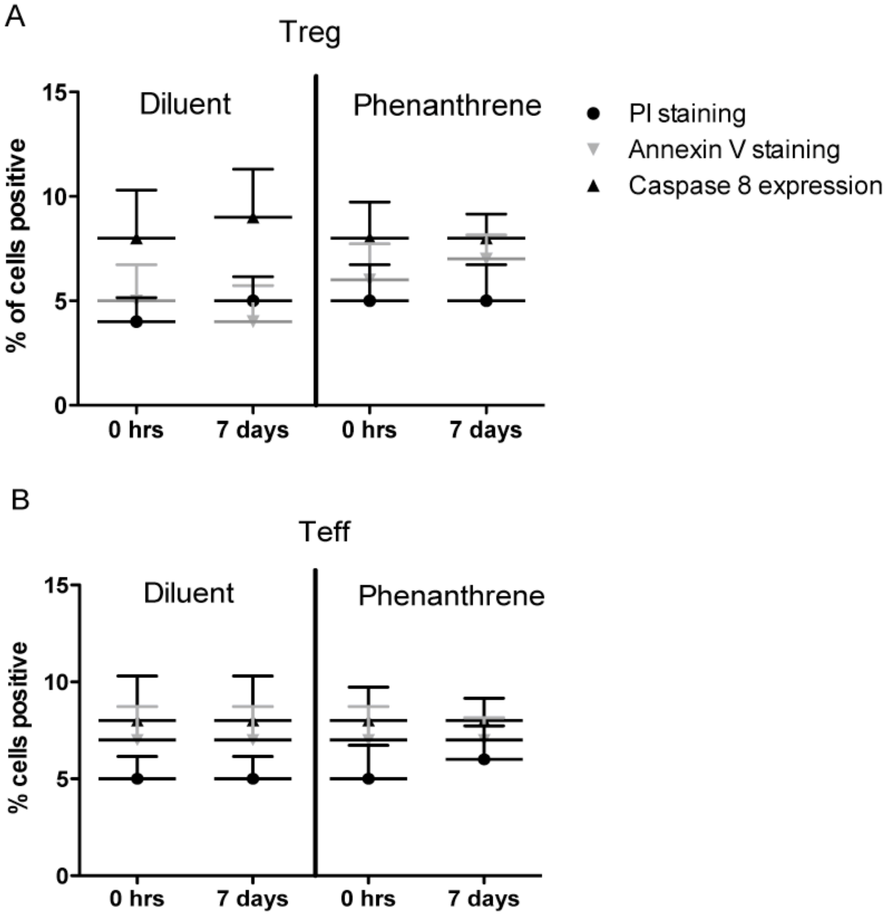

**Figure S6. Phenanthrene exposure results in impaired chemotaxis of Treg to BEC**. A and B) Isolated Tregs were treated with phenanthrene (300 nM, black bars) or diluent for 0 to 7 days and analyzed for expression of chemokine receptors CCR4 and CCR8 by immunostaining and flow cytometry. C) Treg were incubated with phenanthrene (300 nM) for 24 hours and then measured for their ability to migrate to primary human bronchoepithelial cells, as previously published. Chemotaxis indices were generated from number of Treg migrating to human BEC divided by number of spontaneously migrated cells. Error bars show SD.

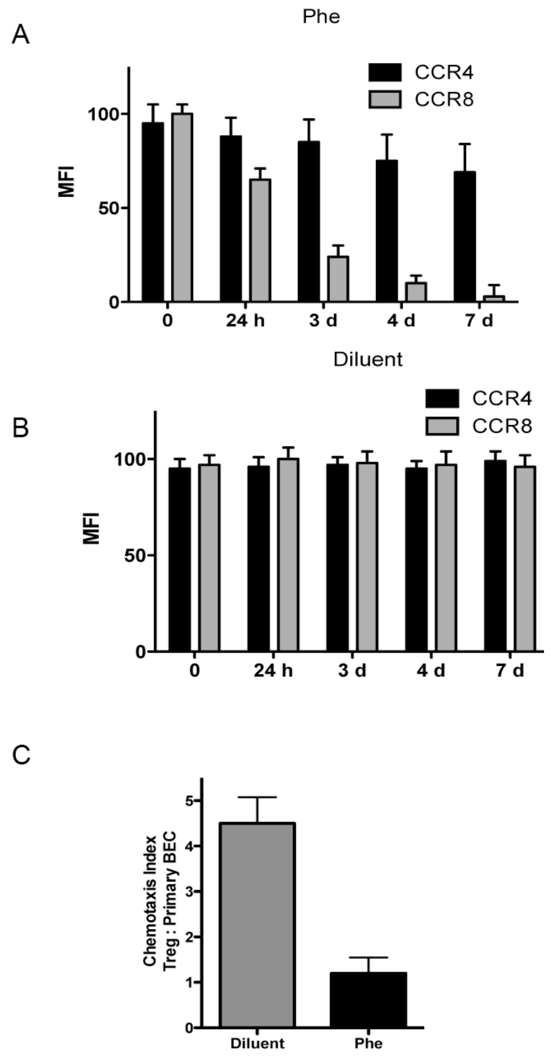

**Figure S7. AhR mediates Foxp3 and DNMT expression in mice.** (A) CD3+CD4+CD25hi Treg (n=3) and (B) splenic CD4+ Teff (n=1) were isolated from AhR+/+ (black bars), AhR+/- (grey bars) and AhR-/- (white bars) mice and analyzed for *Dnmt1, Dnmt3b, Foxp3* and *AhR* transcript expression by QT-PCR. Expression levels were normalized against *Hprt.* Data are expressed as mean plus SD of triplicates run on each sample. * P < 0.05.

A

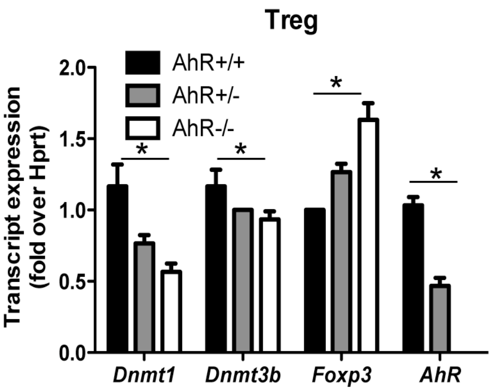


B

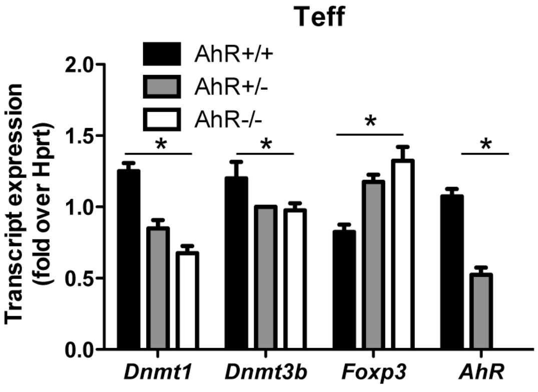

Supplement: Supplementary file 1 — T Cell Isolation and Phenotyping Healthy donor samples were obtained from the Stanford Blood Center and sorting via flow cytometry was performed (FACS Aria, BD Biosciences). Each population obtained from flow cytometry was tested for purity based on multicolor flow cytometry staining for CD4+CD25hiCD127lo (Treg) and CD4+CD25neg (conventional CD4+ T cell). Only samples meeting or exceeding 97% pure were used for further experimentation and analysis. Fluorescently labeled, directly conjugated antibodies were used. Further descriptions are provided in the supplementary material. [file 967029.f1.pdf]
